# Review of Australian health economic evaluation – 245 interventions: what can we say about cost effectiveness?

**DOI:** 10.1186/1478-7547-6-9

**Published:** 2008-05-20

**Authors:** Kim Dalziel, Leonie Segal, Duncan Mortimer

**Affiliations:** 1 Health Economics and Policy Group, Division of Health Sciences, University of South Australia, Adelaide, Australia; 2 Centre for Health Economics, Monash University, Melbourne, Australia

## Abstract

**Background:**

There is an increasing body of published cost-utility analyses of health interventions which we sought to draw together to inform research and policy.

**Methods:**

To achieve consistency in costing base and policy context, study scope was limited to Australian-based cost-effectiveness analyses. Through a comprehensive literature review we identified 245 health care interventions that met our study criteria.

**Results:**

The median cost-effectiveness ratio was A$18,100 (~US$13,000) per QALY/DALY/LY (quality adjusted life year gained or, disability adjusted life year averted or life year gained). Some modalities tended to perform worse, such as vaccinations and diagnostics (median cost/QALY $58,000 and $68,000 respectively), than others such as allied health, lifestyle, in-patient interventions (median cost/QALY/DALY/LY all at ~A$9,000~US$6,500). Interventions addressing some diseases such as diabetes and impaired glucose tolerance or alcohol and drug dependence tended to perform well (median cost/QALY/DALY/LY < A$3,700, < US$5,000). Interventions targeting younger persons < 25 years (median cost/QALY/DALY/LY < A$41,200) tended to perform less well than those targeting adults > 25 years (median cost/QALY/DALY/LY < A$16,000). However, there was also substantial variation in the cost effectiveness of individual interventions within and across all categories.

**Conclusion:**

For any given condition, modality or setting there are likely to be examples of interventions that are cost effective and cost ineffective. It will be important for decision makers to make decisions based on the individual merits of an intervention rather than rely on broad generalisations. Further evaluation is warranted to address gaps in the literature and to ensure that evaluations are performed in areas with greatest potential benefit.

## Background

Because resources are limited not all potentially beneficial services can be funded. Choices must be made in allocating scarce resources. Economic evaluation can help inform resource allocation choices by comparing costs and consequences of two or more alternatives. Comparisons between interventions will be more robust where they are country specific, at least in terms of input costs, which differ considerably between countries. To date Australian economic evaluations have not been systematically described, appraised or explored, except for decisions of the PBAC (Pharmaceutical Benefits Advisory Council) between 1991 to 1996[1]. However, given confidentiality of data, the performance of specific interventions was not reported. There is now a substantial body of published health economic evaluations in Australia that have used 'final and global' measures of performance (life years, quality adjusted life years and disability adjusted life years) which allows comparison across health care interventions.

The aim of the current paper is to describe and explore Australian published economic evaluations and to analyse the distribution of published cost-effectiveness ratios. This analysis will determine whether there is any identifiable pattern in published cost-effectiveness ratios. The results will potentially to assist policy makers with resource allocation decisions and will identify gaps in the types of interventions evaluated.

## Methods

### Searching for cost-effectiveness studies

The Medline OVID database from 1966 to present was searched in April 2005 for relevant studies using key words for "cost effectiveness" and "economic evaluation" combined with the key word "Australia". In addition websites of Australian health economics centres and government health departments were searched [See Additional file 1]. Key words such as "cost", "economic" and "evaluation" were used separately. Bibliographies of the articles reviewed were searched for further relevant articles, and a key author search was conducted for authors identified with multiple relevant publications. No restrictions were made by year of publication, and all publicly available reports and papers were eligible for inclusion.

### Selection

Studies of economic analysis of lifesaving or quality enhancing "health" interventions were eligible for inclusion, defined as broadly fitting within the context of the health care system. An initial selection of potentially relevant articles was made by one reviewer (KD). This selection was broad and overly inclusively.

The following inclusion criteria were then applied by two reviewers (KD and DM) independently to each full text article initially identified as potentially relevant. Consensus was reached by discussion.

• Resources were estimated in Australian dollars.

• The economic evaluation presented as cost per LY saved, death averted, QALY gained or DALY averted, or this could be simply calculated from the figures provided.

• The article was published in English.

• The article was not a duplicate publication. The most complete or recent work by the authors was selected for inclusion with supplementary information retrieved from other reports. Publication on similar interventions by different authors did not class as duplicate.

• The study was primary research. Review articles citing the work of others were excluded, although the reference lists were searched for additional relevant publications.

### Validity assessment

An assessment of the quality of the economic evaluations was performed by one reviewer (KD) following study inclusion. The quality criteria reflect items taken from a framework for quality of cost-effectiveness models developed by Sculpher et al[2]. This instrument was chosen as it incorporates economic modelling as well as evaluation and is therefore broader in scope than other quality appraisal checklists that only apply to economic evaluation. The Sculpher framework provides a list of dimensions of a quality economic model and what constitutes good practice. In addition a list of questions is provided in order to enable the framework to be used as a practice tool for critical appraisal. There are a number of dimensions to the framework including structure, disease states, options (comparators), time horizon, cycle length, data identification, data incorporation, internal and external consistency. The items deemed most appropriate for our brief appraisal were taken from the categories 'options', 'data identification' and 'data incorporation'.

The strength of underlying evidence was rated strong (RCT or meta-analysis) or limited (not RCT or meta-analysis). The comparator chosen for the evaluation was rated either as appropriate (described and justified) or inappropriate (not described or justified). Measurement of costs was rated as appropriate (marginal, clearly described, sources of price and quantity data cited) or inappropriate. Each evaluation was rated as having sensitivity analysis performed or not performed.

### Data abstraction

Where articles included analysis of more than one intervention, data were extracted for each separate intervention. Data abstraction was performed by one reviewer (KD) with checking of key variables by a second reviewer (LS). The following types of variables were extracted: the characteristics of the target disease and patients, details of the intervention, nature of publication and study methodology, and estimated performance. Policy relevant variables, including funding status were separately ascertained (Table 1). These variables were chosen for their possible relationship to cost effectiveness, based on the author's knowledge of the literature and their experiences with priority setting exercises.

**Table 1:** Details of variables extracted

**Type of variables**	**Variables extracted (See also table 3)**
Nature of Publication	Type of publication, Source of publication, Type of journal.
Target of intervention (eg Patient characteristics)	DRG, Age, General vs. specific population, Ability to reduce own risk of disease/death (eg obesity reduction), Condition caused by own behaviour (eg smoking related)
Intervention characteristics	Year, Type of program (medical vs lifestyle), Prevention stage, Intervention objective (eg treatment, diagnosis, screening), Modality (pharmaceutical, primary/specialist medical care, community/media/education, hospital inpatient, vaccination, allied health, other).
Methodology	Type of evidence, Level of evidence, Economic perspective, Type of evaluation, Discount rate, Time horizon for model, Duration of benefit for model, Appropriateness of comparator, Appropriateness of cost measurement, Use of sensitivity analysis.
Cost effectiveness	Cost per LY/QALY/DALY, Intervention dominated or dominant.

### Data synthesis

Cost per LY/QALY/DALY estimates were reviewed and recalculated where necessary to ensure each referred to marginal costs and benefits. Estimates were standardised by translating values into June 2005 estimates using the health component of the CPI[3]. If a study reported a range for the cost-effectiveness results, the study was examined to determine if different estimates related to different interventions and/or distinct target populations. If this was the case, the cost-effectiveness ratio for each distinct population and/or intervention was extracted. However where such sub-groups were the result of post hoc analysis not consistent with delivery of the intervention a standardised figure across all groups was calculated using Australian population data (eg proportion male/female in target age group). If the range simply represented upper and lower limits from sensitivity analyses, a central estimate was used where reported or calculated as the mean if not.

In the event that a reference year was not reported for costs, we used the publication year minus two to reflect the usual delay in publishing original research. Categorisation of the type of intervention, type of patients and results was possible for all studies included in the review. The only sources of missing data were discount rate, time horizon and length of intervention benefit which were purely descriptive variables.

### Analysis

Data were described using medians and interquartile ranges for continuous data and proportions for categorical data. The pattern of cost-effectiveness results across the 245 interventions was explored through a combination of descriptive and regression analyses. Ordinary least squares regression was undertaken to identify variables that might explain variation in the cost per LY/QALY/DALY estimates. Ordered logit regression was undertaken to identify variables that might explain variation in the cost per LY/QALY/DALY group. All regressions adjusted for intra-cluster correlation present in the data because data on multiple interventions were drawn from many of the papers included in our review. We used the robust Huber/White sandwich estimator to adjust population-average models for intra-cluster correlation, yielding robust standard errors suitable for calculating confidence intervals around estimated regression coefficients[4].

All potentially relevant intervention and publication characteristics listed in Table 2 were initially included in the regression and retained on the basis of their contribution to the regression as evaluated by t- and F-tests (enter p ≤ 0.05) for individual and joint significance, with care taken to ensure stability in the magnitude and direction of the beta coefficients when adding or dropping a potentially relevant variable. Collinearity between included variables and potentially relevant variables excluded from the regression was investigated using standard diagnostics and by methodically entering, removing and re-entering combinations of variables. Results were confirmed by examining outputs from backwards and forwards stepwise regression analyses as evaluated by the probability of F (enter p ≤ 0.05, remove p ≥ 0.10).

**Table 2:** Descriptive statistics of the 245 interventions

**No. Interventions (%)Total 245**		
**Patient/disease characteristics**		

AR-DRGs	Musculoskeletal and connective tissue	33 (13)
	Mental diseases and disorders	32 (13)
	Alcohol or drug use	29 (12)
	Circulatory system	22 (9)
	Endocrine nutritional and metabolic disorder/disease	20 (8)
	Infectious and parasitic diseases	20 (8)
	Other	89 (36)
Target Age	Children aged 0 to 14 years	34 (14)
	Young adults age 14 to 25 years	5 (2)
	Working age adults 25 to 65 years	14 (6)
	Elderly aged 65+ years	20 (8)
	Children and young adults aged 0 to 25 years	2 (1)
	Young adults and adults aged 14 to 65 years	63 (26)
	Adults and elderly aged 25 to 65 plus years	92 (38)
	All	11 (5)
	Mixture of the above groups	4 (2)
Target population	Specific	200 (82)
	General population	45 (18)
Ability to reduce own risk	To some extent	123 (50)
	No	122 (50)
Condition caused by patients' own behaviour	To some extent	127 (52)
	No	118 (48)

**Intervention details**		

Type	Medical	177 (72)
	Lifestyle	68 (28)
Objective	Treatment	119 (49)
	Prevention	78 (32)
	Screening	33 (14)
	Diagnosis	7 (3)
	Combination	8 (3)
Prevention stage	Primary (completely avert disease)	78 (32)
	Secondary (slow/halt progression of disease)	119 (49)
	Tertiary (limit disability after harm)	48 (20)
Modality	Pharmaceutical	52 (21)
	Primary medical care or specialist care	65 (27)
	Community/media/education	39 (16)
	Hospital inpatient	26 (11)
	Vaccination	17 (7)
	Allied health	29 (12)
	Combination of modalities	9 (4)
	Other	8 (3)

**Nature of publication & study methodology**		

Year of publication	Median (range)	2002 (1989 to 2005)
Strength of evidence	Strong-RCT/meta-analysis	132 (54)
	Limited	113 (46)
Where published	Peer reviewed journal	185 (76)
	Government report	18 (7)
	Other peer-reviewed report	26 (11)
	Other non peer reviewed report	16 (7)
Publication type	General medicine	52 (21)
	Specialist medicine	85 (35)
	Health economics/policy/HTA/public health	108 (44)
Perspective	Health system	212 (87)
	Societal	33 (14)
Measure of Outcome	Life year	79 (32)
	QALY	119 (49)
	DALY	46 (19)
	HYE	1 (1)
Discount rate	0%	27 (13)
	3%	22 (10)
	5%	190 (78)
	Missing	12 (6)
Time horizon of the model in years	Median	15
	Interquartile range	1 to 25
	Missing	89 (36)
Duration of benefit in years	Median	4
	Interquartile range	1 to 6
	Missing	81 (33)
Downstream costs/savings	Included	140 (57)

**Quality**		

Q-Comparator	Appropriate	215 (88)
Q-Costs	Appropriate (marginal and clear)	185 (76)
Q-Sensitivity analysis	Performed	239 (98)
Q-Overall	Met all three requirements above	173 (71)

**Cost effectiveness**		

	More effective but more costly	214 (87)
	Median	$19,017
	Interquartile range	$5,997 to $45,670
	Dominated	11 (5)
	Dominant	20 (8)

**Funding & implementation**		

Funding status	Fully funded	87 (35)
	Partially funded	75 (31)
	Not funded	83 (34)
Patients required to make contribution to costs	Yes	178 (73)

## Results

### Trial flow

**Figure 1:**
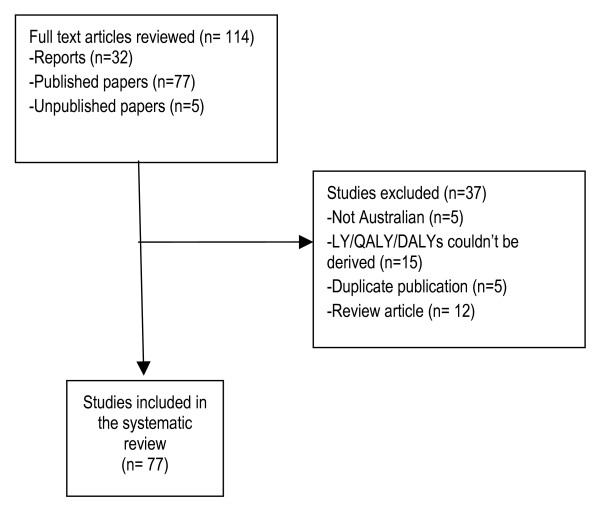
Description of study flow.

The Medline search lead to 912 results, of which 42 (4.6%) were identified as potentially relevant through screening titles and abstracts. An additional 11 papers or reports were identified through key author searches, 9 through reviewing bibliographies of identified articles and 52 through the website searches. A total of 114 full text documents were examined for inclusion in this review (Figure 1 describes the exclusion process) with a total of 77 (68%) included.

### Descriptive results

Of the 77 included documents, sufficient information was available to calculate cost per QALY, DALY or LY estimates for 245 interventions. Table 2 summarises the patient/disease characteristics, intervention details, methodology, quality and implications related to these 245 interventions.

### Cost effectiveness

**Figure 2:**
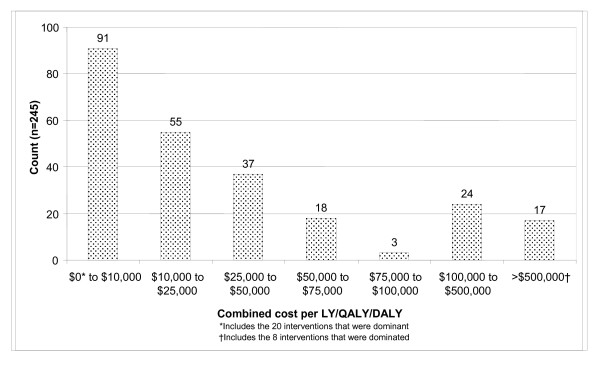
Number of interventions in each cost per LY/QALY/DALY group category (A$).

For studies reporting LYs, the median value was A$18,720 per LY gained and for those reporting QALYs or DALYs, the median value was A$17,830 per QALY/DALY. The median economic performance using QALYs/DALYs where available or LYs otherwise across all 245 interventions was A$18,100 per LY/QALY/DALY. Eleven interventions (5%) were more costly and less effective than their comparators and were therefore dominated, 21 interventions (8%) were both more effective and cheaper than their comparator and thus dominant. Figure 2 illustrates the distribution of incremental cost per LY/QALY/DALY ratios. A large number of interventions (n = 91, 37%) reported ICERS that were less than A$10,000 per LY/QALY/DALY, (including the 8% that were dominant). One hundred and forty-six interventions (60%) reported ICERs that were less than A$25,000 per LY/QALY/DALY. A further 41 interventions (17%) were reported with an incremental cost of greater than A$100,000 per LY/QALY/DALY (including the 5% that were dominated).

Table 3 presents the median, 25^th ^and 75^th ^percentile cost-effectiveness ratio of each category and reports statistical significance. Statistically significantly higher median incremental cost-effectiveness ratios (ICERs) (performed worse) were found for interventions targeted at children/youth compared to adults, for medical interventions compared with lifestyle interventions, vaccinations compared to all other modalities, evaluations where downstream cost impacts were not included. In relation to quality variables, the small number of evaluations that did not use an appropriate comparator and did not meet minimum standards of quality performed better. Evaluations based on strong quality evidence (strength of evidence) with regards to treatment effect were associated with similar median cost-effectiveness estimates as evaluations with limited quality evidence. Those that were associated with statistically significantly lower median ICERs, included the following:

**Table 3:** Cost per QALY/DALY/HYE by patient/disease characteristics, intervention characteristics, methodological attributes, quality of study, funding of intervention

**Variable name**	**Categories**	**ICER**			**χ^2^, p-value^a^**
		**25^th ^percentile**	**50^th ^percentile (median)**	**75^th ^percentile**	

Target population	General population	$8,798	$20,449	$150,496	
	Specific (targeted high risk group)	$2,392	$17,220	$45,068	2.446, 0.118
Age group	0 to 25 years	$11,586	$41,195	$149,581	
	> 25 years	$2,370	$15,927	$42,801	**8.903, 0.003**
Type of intervention	Medical eg physician consult, pharmaceuticals, vaccinations, diagnostic tests, inpatient visits	$5,946	$21,898	$57,363	**8.247, 0.004**
	Lifestyle eg advice to alter diet/physical activity	$1,678	$10,015	$24,920	
Modality1	Pharmaceutical	$11,781	$26,871	$53,986	
	All else (primary/specialist care, vaccination, allied health, community/media/education, inpatient)	$2,232	$15,270	$44,558	2.787, 0.095
Modality2	Allied health, community/media/education	$1,899	$9,591	$31,749	**4.609, 0.032**
	All else	$5,507	$20,449	$57,363	
Vaccination	Vaccination	$12,625	$56,408	$156,400	
	All else	$2,642	$17,827	$44,711	**4.310, 0.038**
Objective of intervention	Treatment (eg cox2 inhibiters to ameliorate symptoms of osteoarthritis)	$2,045	$14,161	$38,620	**2.275, 0.131**
	All else (prevention, screening, diagnosis, combination)	$4,674	$20,650	$58,817	
Disease stage	1) Treatments designed to completely avert disease/injury or slow, halt or reverse progression of disease/injury (primary and secondary prevention)	$2,514	$17,827	$43,805	2.534, 0.111
	2) Treatments designed to limit disability after harm has occurred (tertiary prevention)	$6,048	$19,310	$133,284	
Ability to reduce own risk of disease/injury	To some extent (eg heart disease)	$1,671	$13,778	$32,644	
	No	$7,679	$25,747	$111,031	**14.723, < 0.001**
Condition caused by patients' own behaviour	To some extent (eg liver cirrhosis)	$1,664	$13,311	$25,894	
	No	$10,299	$29,609	$100,871	**24.001, < 0.001**
Year	Pre-1993^b^	$1,698	$6,259	$97,213	
	1993^† ^to 1997^c^	$11,367	$34,820	$81,629	
	Post-1997^c^	$3,436	$17,616	$43,769	3.177, 0.204
Strength of evidence	Strong – RCT and/or meta-analysis	$3,524	$18,282	$44,794	
	Limited – other study design	$2,356	$18,039	$56,608	0.072, 0.788
Perspective	Health system	$2,642	$17,613	$46,738	
	Societal	$8,122	$20,165	$75,116	1.335, 0.248
Outcome	Life year		$18,724		
	QALY/DALY/HYE		$17,827		0.080, 0.777
Discount rate	< 5%	$7,981	$25,747	$43,761	
	= 5%	$2,407	$15,553	$54,028	**0.436, 0.509**
Downstream costs/savings	Included	$1,846	$13,871	$40,658	
	Not	$6,897	$21,405	$59,317	**3.866, 0.049**
Q-Comparator	Appropriate	$6,437	$20,891	$58,318	
	Not	$983	$2,257	$6,409	**27.392, < 0.001**
Q-Costs	Appropriate (marginal and clear)	$3,802	$21,885	$54,483	
	Not	$2,045	$12,706	$25,082	3.554, 0.059
Q-Sensitivity	Performed	$3,189	$18,360	$51,583	
	Not	$1,695	$10.895	$30,736	0.756, 0.385
Q-Overall	Adequate	$4,406	$22,437	$56,395	
	Not	$2,221	$14,082	$22,887	**5.111, 0.024**
Funding status	Fully funded	$5,735	$20,165	$53,558	
	Partially funded	$1,358	$9,011	$35,429	**10.870, 0.004**
	Not funded	$6,351	$20,850	$97,378	
Patients required to contribute to costs	Yes (eg co-payment for pharmaceuticals)	$3,789	$18,724	$43,769	0.035, 0.852
	No (eg immunisations provided free of charge)	$7,981	$15,733	$110,806	

a) Statistical significance was assessed using the median value and the Kruskall-Wallace H test for independent samples, all degrees of freedom were equal to one with the exception of the test for year of publication where df = 2, statistically significant results are highlighted in bold

b) From January 1993, the PBAC required to take into account cost effectiveness when making recommendations for listing.

c) Establishment of the Medical Services Advisory Committee (MSAC) in 1998.

• non-medical interventions (allied health community, media, education) compared to medical (physician consult, pharmaceutical, in-patient, vaccinations),

• treatment interventions compared to diagnosis/screening/prevention,

• interventions where the individual was able to reduce their own risk of disease or injury,

• interventions where the condition was cause by patients' own behaviour, and

• interventions that were partially funded (some government subsidy but not to meet all clinical need) rather than fully or not funded all.

**Figure 3:**
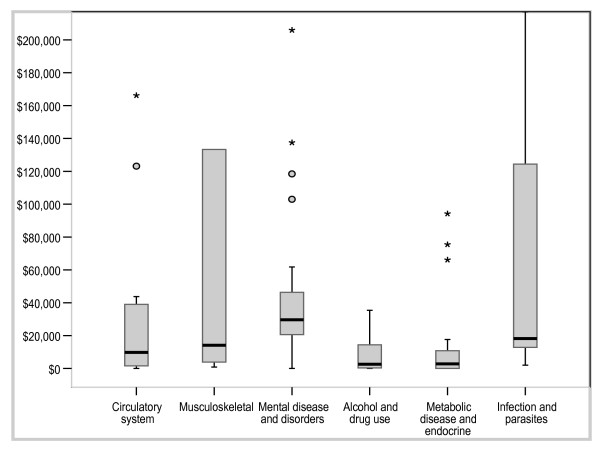
Distribution of cost per LY/QALY/DALY by selected major diagnostic groups (A$).

**Figure 4:**
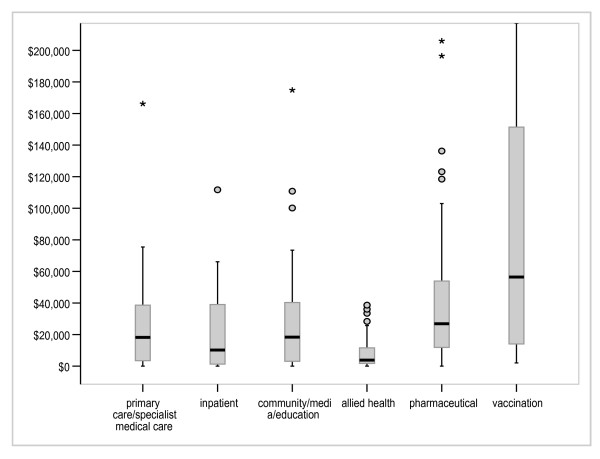
Distribution of cost per LY/QALY/DALY by modality (A$).

**Figure 5:**
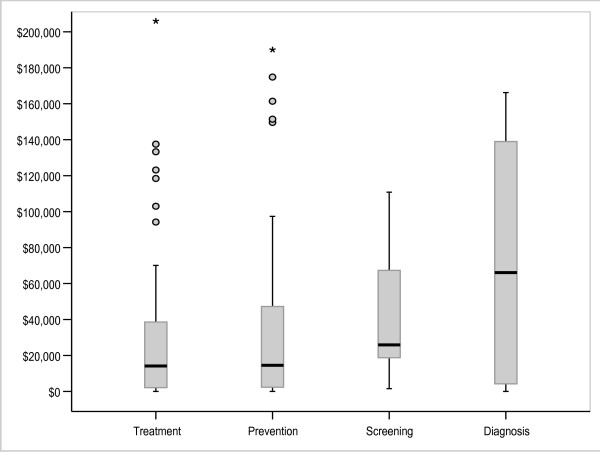
Distribution of cost per LY/QALY/DALY by objective of intervention (A$).

Figures 3, 4 and 5 illustrate results for the variables modality, objective and type of disease (DRG). Diagnostic tests were associated with higher cost-effectiveness ratios and greater variation than were screening, treatment and prevention. Because there were small numbers of interventions in some DRG groups, we have reported on the 6 DRG groups containing a sufficient number of interventions for meaningful between-group comparisons. The cost-effectiveness ratios varied across DRG groups with the 'alcohol and drug use' and 'metabolic disease' categories having relatively little variation around a particularly low median cost-effectiveness ratio, the 'mental disease/disorder' group having the highest median cost-effectiveness ratio and the 'musculoskeletal' and 'infection groups' having the most variability. Examining modality; pharmaceuticals and vaccinations had higher and more varied cost-effectiveness ratios than other modalities, whilst allied health interventions and inpatient care had the lowest median cost-effectiveness ratios.

That said, the extent of variation in the data is such that there were examples of highly cost-effective and cost-ineffective care within most categories.

### Exploring determinants of cost effectiveness

Linear regression analysis using the enter method was undertaken to identify variables from those listed in Table 2 that might explain variation in the cost per LY/QALY/DALY estimates. The intra-cluster correlation coefficient for cost per LY/QALY/DALY (ICC = 0.332, 95%CI: 0.17, 0.49) suggested that some adjustment should be made for clustering by paper in this analysis. Table 4 summarises parameter estimates, model fit and individual significance of included variables from the Huber/White sandwich estimator. MODALITY 1 (pharmaceutical = 1 versus all else = 0), REDUCE RISK OF DEATH/DISEASE (yes = 1 versus no = 0), AGE (25 to 65 years = 1 versus all else = 0), PATIENT CONTRIBUTION TO COSTS (yes = 1 versus no = 0) and Q-SENSITIVITY (sensitivity analysis performed = 1 versus not = 0) were significant predictors but explained just 1.5% of variance in economic performance as measured by cost per LY/QALY/DALY. MODALITY 1 was the most important independent variable based on the size of the beta coefficient (β = -129,593, p = 0.038). Ramsey's Reset Test for the presence of omitted variables in the residuals was insignificant (F(3,236) = 0.18, p = 0.909), suggesting that the majority of between-intervention variation in cost per LY/QALY/DALY ratios is random.

**Table 4:** Parameter estimates and model fit for OLS regression on cost per LY/QALY/DALY

Predictor	β	Robust SE	t	Sig.	R^2^
(Constant)	160,830	99,591	1.61	0.111	-
AGE	-211,012	40,081	-3.04	0.003	0.0035
Q-SENSITIVITY	-108,172	51,011	-2.12	0.037	0.0043
MODALITY1	-129,593	58,881	-2.20	0.031	0.0071
REDUCE RISK^	125,527	59,489	2.11	0.038	0.0101
PATIENT CONTRIBUTION^	106,676	64,718	1.65	0.104	0.0150

^Jointly significant at 0.05 level (F(2, 73) = 5.70, p = 0.005).

Interpretation of the parameter estimates is straightforward. Pharmaceuticals (compared to non-pharmaceuticals) and interventions primarily benefiting persons aged between 25 and 65 years would generally have a lower cost per LY/QALY/DALY than an intervention benefiting older or younger age groups. The quality of evaluation also made a significant contribution to the regression such that a failure to conduct sensitivity analysis was associated with a lower cost per LY/QALY/DALY ratio. Interventions targeting persons able to reduce their own risk of death/disease and interventions that are partially funded out of patient contributions would also generally have a higher cost per LY/QALY/DALY than otherwise. It is, however, important to note that the regression explains only a small proportion of the overall variance in cost per LY/QALY/DALY group.

We also undertook an ordered logit regression to identify variables from those listed in Table 2 that might explain variation in economic performance expressed in terms of cost per LY/QALY/DALY group. The intra-cluster correlation coefficient for cost per LY/QALY/DALY group (ICC = 0.392, 95%CI: 0.23, 0.56) suggested that some adjustment should be made for clustering by paper in this analysis. Table 5 summarises parameter estimates, model fit and individual significance of included variables from the Huber/White sandwich estimator. TARGET (general population = 1 versus specific = 0), DISEASE STAGE (limit disability after harm has occurred = 1 versus avert, slow or halt disease or injury = 0), CAUSED BY (patient's own behaviour contributed to condition = 1 verus not = 0) DOWNSTREAM (downstream costs/savings = 1 included versus not = 0), NOT FUNDED (not funded = 1 versus fully or partially funded = 0) and Q-OVERALL (adequate comparator, costs and sensitivity analysis = 1 versus not = 0) were significant predictors but explained just 8.1% of variance in economic performance as expressed in terms of cost per LY/QALY/DALY group. CAUSED BY was the most important independent variable based on the size of the beta coefficient (1.4, P < 0.001).

**Table 5:** Parameter estimates and model fit for ordered logit regression on cost per LY/QALY/DALY group

Predictor	β	Robust SE	Z	Sig.	R^2^
TARGET	0.693	0.265	2.61	0.009	0.003
DISEASE STAGE^	0.904	0.543	1.67	0.096	0.006
CAUSED BY	1.545	0.230	5.15	0.000	0.035
DOWNSTREAM^	-0.561	0.302	-1.86	0.063	0.039
NOT FUNDED	0.956	0.267	3.58	0.000	0.051
Q-OVERALL	1.378	0.395	3.49	0.000	0.081

Thresholds	β	Robust SE			

Dominant to $10 K = 1					
$10 K to $25 K = 2	1.383	0.470			
$25 K to $50 K = 3	2.500	0.491			
$50 K to $75 K = 4	3.363	0.560			
$75 K to $100 K = 5	3.902	0.596			
$100 K to $500 K = 6	4.005	0.607			
> $500 K = 7	5.111	0.771			

^Jointly significant at 0.05 level (Wald χ^2 ^= 6.43, p = 0.040).

## Discussion & conclusion

Through this study, data are now available on the economic performance, expressed in Australian costs, of a wide range of interventions that address different health problems, using alternative modalities and intervening at various stages in disease development. The identification of a large number of interventions (37%) reported at less than A$10,000 per LY/QALY/DALY (including 8% that were dominant), which is below any putative funding threshold is important in itself. It raises issues about the relationship between cost effectiveness and funding decisions and the appropriateness of current funding thresholds. These matters are explored elsewhere[5].

We identified some interesting findings by category, for example that interventions targeted at children were generally less cost-effective than those targeting adults. This is perhaps not surprisingly, especially in relation to chronic disease prevention where benefits are typically delayed at least into middle age. Similarly, 'population approaches' were not found to be more cost effective than more targeted approaches, which may reflect very large differences in effectiveness. It would be interesting to explore the especially good and especially poor performance of some classes of intervention; such as the poor performance of diagnostics and vaccinations or the favourable performance of allied health and lifestyle interventions and those addressing diabetes and drug/alcohol abuse. That said category averages should be interpreted with care due to the identified wide variation in cost effectiveness with no 'magic bullet' answers to resource allocation. In terms of policy decision it would be best to assess each potential intervention on its own merits rather than rely on broad generalisations [6-10].

We also note that this is the first review of publicly available Australian economic evaluations, which provides valuable information to guide policy and research, but also highlights the continued need for improvement in quality of economic evaluation and transparency. This type of exercise, summarising the cost effectiveness of different interventions and subgroups has been proposed as a useful priority setting task [11], with precedents in the United States[12,13]. This review, in summarising all the published Australian economic evaluations also provides a platform for investigating where evaluations have been targeted and what this says about implicit priorities. It also allows an exploration of the distribution of cost-effectiveness ratios relative to funding thresholds and an analysis of the quality of evaluations. From this work we can for instance map the areas subject to economic evaluation in Australia against the existing burden of disease, and assess the scope of coverage of modalities and delivery settings to check for alignment of research priorities. In order to achieve system wide allocative efficiency in health care, information is required across a broad range of interventions, considering target diseases, age groups, disease stage, modality and delivery settings.

The limitations of this review include a reliance on publicly available evaluation reports. While it is possible that some studies were missed through our original search focus on Medline, a later search of the HEED (NHS Economic Evaluation Database, Cochrane Library) database using the same search terms identified no additional studies.

With regards to quality, this review has inherited the quality of the original work, which we have attempted to describe. Interestingly, the pattern of cost effectiveness of interventions where evaluations were based on limited non-RCT evidence did not differ from those based on stronger RCT evidence. There is no reason to presume that potential biases will systematically impact on cost-effectiveness results.

A significant limitation of this work is that the economic evaluation methods varied significantly between interventions thus impacting on the comparisons made. This is illustrated in identified differences in discounting, perspective, time horizons, choice of comparators and strength of underlying evidence. The strength of this work therefore lies in the rich description of existing evaluations. Ideally all outcome measures would be identical to assist with comparisons. However, we would contend that there is enough common ground between the outcome measures QALY, DALY and LY for cost-effectiveness ratios to be sensibly compared. Evaluations reporting cost per LYs gained may have generally focused on length of life because quality of life was not expected to vary greatly relative to the impact on mortality. Despite differences in the concept of 'health' underlying adjustments for morbidity using the QALY or the DALY, these do include both mortality and morbidity effects. However, we acknowledge that this is a potential source of error. We were limited in that study resources only permitted one person to perform data extraction of variables. This is unlikely to have lead to bias against single interventions or group of interventions, but may have involved a particular interpretation of variables extracted across all studies.

The list of interventions and associated cost-effectiveness ratios is reported [See Additional file 2](the authors would be pleased to provide a copy of the full database on request). However, the use of these cost-effectiveness results as a strict league table was not the intended purpose of this exercise; rather this work was intended as a broader information resource for research and policy. The review is not a complete priority setting tool as it does not include all potentially important interventions and in that context, methodological differences between studies that we have drawn on are important.

### Relation to previous research

The cost effectiveness of Australian Pharmaceuticals has been previously reported in a review of PBAC (Pharmaceutical Benefits Advisory Council) decision making from 1991 to 1996[1]. Twenty-six submissions were analysed with a median cost per LY of A$43,550 ($1998/1999) which is higher than the median estimate for pharmaceuticals reported here of $A22,000 ($2005). The interventions were all drawn from submission by the pharmaceutical companies to the PBAC. Companies have a vested interest in these evaluations which are used both to inform whether a drug will be listed on the PBS (Pharmaceutical Benefits Schedule) and subsidized by government and the approved price. This creates an incentive to report a cost/LY just below the apparent funding threshold, which on the basis of funding decisions would seem to lie within the range of $A40,00 to $A70,000/LY or/QALY[1]. Our sample of pharmaceuticals was also larger than the previous sample. However, it is also true that the cost-effectiveness profile will depend on the actual list of interventions included, with individual results also impacted by any 'agendas' of the researchers. For this reason our review was limited to reports in the public domain.

In the US a large scale review exercise was undertaken of 500 life saving interventions across the areas of health, transport and environment[14]. Tengs et al[14] reported a median medical intervention cost of 1993 US$19,000 per life year, with wide variation, which is not dissimilar to our median estimate of A$18,100 (US$15,400 based on exchange rates 30 June 2007), even allowing inflation to current values, given the cost difference between the US and Australian health care systems. Tengs et al[14] reported a lower cost-effectiveness ratio for primary prevention medical interventions of US$5,000 compared to US$23,000 for secondary and US$22,000 for tertiary prevention. This compares with our estimates of A$14,900 (US$11,100) for primary prevention, A$18,200 (US$13,500) for secondary and A$28,800 (US$21,400) for tertiary prevention, using the same definitions (note that dollars are standardised to 2005 for our work but to 1993 for the Tengs et al[14] review).

A more recent review was conducted of cost-utility analyses in the United States (494 studies and 1433 cost-effectiveness ratios)[12,13,15]. The results of this review are also comparable with a median ICER of US$20,133 (with dollar estimates taken from studies covering 1976 to 2001 – unadjusted and non-standardised).

Published cost-effectiveness results may reflect the research interests or priorities of researchers or industry, the visibility of certain diseases, the strength of advocacy and industry backing rather than the health needs of society[13]. The results of this review identify implicit priorities. Knowing where economic evaluations have been focused in the past, it would be useful to determine where cost-effectiveness efforts in Australia are likely to yield the greatest benefit. A more coordinated approach to health economic evaluation may lead to a better coverage of the priority health areas and important interventions and could also be used to encourage greater consistency in results, aiding comparability.

### Implications for further research

This exercise should be repeated for other countries, as findings are likely to vary according to the delivery arrangements and costing structure of different health systems. There is the opportunity using datasets such as this for a more in depth analysis of the quality of economic evaluation, which could be used to inform evaluator training and guide methodological advances. It would also be possible to compare the quality of evaluations over time to assess improvements.

Another application of this work is to explore the extent to which economic evaluation informs policy making. Our recent extension to this work [5] addresses some of the issues concerning the funding of interventions including an exploration of the characteristics of interventions that are related to a higher chance of funding at particular cost-effectiveness thresholds. This provides evidence of the apparent success of current priority setting arrangements in guiding the health sector towards a more efficient allocation of resources across modalities and across disease-stage [16].

## Abbreviations used

A$: Australian Dollar; ABS: Australian Bureau of Statistics; AIHW: Australian Institute of Health and Welfare; AR-DRG: Australian refined diagnosis-related group; CPI: consumer price index; DALY: disability-adjusted life year; DM: Duncan Mortimer; DRG: diagnosis-related group; HEED: NHS Economic Evaluation Database; HYE: healthy-year equivalent; HTA: health technology assessment; ICC: intra-cluster correlation coefficient; ICER: incremental cost-effectiveness ratio; KD: Kim Dalziel; LS: Leonie Segal; LY: life-year; MSAC: Medical Services Advisory Committee; PBAC: Pharmaceutical Benefits Advisory Committee; PBS: Pharmaceutical Benefits Schedule; RCT: randomised controlled trial; SD: standard deviation; US: United States of America; US$: United States Dollar.

## Competing interests

The authors declare that they have no competing interests.

## Authors' contributions

KD participated in the design of the study, coordinated the data collection, participated in the data analysis and interpretation of results, and drafted the manuscript. LS participated in the design of the study and data collection, and suggested edits and revisions to the manuscript. DM participated in the data collection, data analysis and interpretation of results, and suggested edits and revisions to the manuscript. All authors read and approved the final manuscript.

## Supplementary Material

Additional file 1 APPENDIX 1_CERA. A copy of the first appendix detailing the search strategy used in the systematic review Click here for file

Additional file 2 APPENDIX 2_CERA. A copy of the second appendix which provides a list of the 245 Australian health interventions included in the systematic review along with an estimate of their cost effectiveness. Click here for file

## References

[B1] George B, Harris A, Mitchell A (2001). Cost-effectiveness analysis and the consistency of decision making: evidence from the Pharmaceutical Reimbursement in Australia (1991 to 1996). Pharmacoeconomics.

[B2] Sculpher M, Fenwick E, Claxton K (2000). Assessing quality in decision analytic cost-effectiveness models: A suggested framework and example of application. Pharmacoeconomics.

[B3] Australian Bureau of Statistics (2005). 52060 Australian National Accounts: National Income, Expenditure and Product.

[B4] Greene WH (1993). Econometric Analysis.

[B5] Segal L, Dalziel K, Mortimer D (2007). Review of Australian Economic Evaluation in Health: Time to look at the bigger picture – the role of the funding environment. Health Econ.

[B6] Australian Institute of Health and Welfare GP Prevention better than cure says new report.

[B7] Gandjour A, Wilhelm Lauterbach K (2005). Does prevention save costs? Considering deferral of the expensive last year of life. J Health Econ.

[B8] Godfrey PO, Johnston RB (2004). Balancing benefits and harms in public health prevention programmes mandated by governments. BMJ.

[B9] Epstein LH, Valoski AM, Kalarchian MA, McCurley J (1995). Do children lose and maintain weight easier than adults: a comparison of child and parent weight changes from six months to ten years. Obes Res.

[B10] Cohen DR (1990). Introducing quality into cost effectiveness. Int J Qual Health Care.

[B11] Cookson R, McDaid D, Maynard A (2001). Wrong SIGN, NICE mess: is national guidance distorting allocation of resources?. BMJ.

[B12] Bell CM, Urbacj DR, Ray JG, Rosen AB, Greenberg D, Neumann PJ (2006). Bias in published cost effectiveness studies: systematic review. BMJ.

[B13] Neumann PJ, Rosen AB, Greenberg D, Olchanski V, Pande R, Chapman RH, Stone PW, Ondategui-Parra S, Nadai J, Siegel JE, Weinstein MC (2005). Can we better prioritize resources for cost-utility research?. Med Decis Making.

[B14] Tengs TA, Adams ME, Pliskin S, Safran DG, Siegel JE, Weinstein MC, Graham JD (1995). Five-hundred life-saving interventions and their cost-effectiveness. Risk Anal.

[B15] Neumann PJ, Stone PW, Chapman RH, Sandberg EA, Bell CM (2000). The quality of reporting in published cost-utility analyses, 1976–1997. Ann Intern Med.

[B16] Segal L, Mortimer D (2006). A population-based model for priority setting across the care continuum and across modalities. Cost Effectiveness and Resource Allocation.

