# Supplementary material for: Review of Australian health economic evaluation – 245 interventions: what can we say about cost effectiveness?
Source: Cost Eff Resour Alloc. 2008 May 20;6:9. doi: 10.1186/1478-7547-6-9 (PMC2413209; doi:10.1186/1478-7547-6-9)
Supplement: Additional file 1 — APPENDIX 1_CERA. A copy of the first appendix detailing the search strategy used in the systematic review [file 1478-7547-6-9-S1.doc]

APPENDIX 1

Search Strategy

OVID Medline 1966 to present- Searched April 2005

(Cost-benefit analysis/ OR economic evaluation.mp OR cost effective$.mp OR Quality-Adjusted Life Years/ OR cost utility.mp OR models, economic/ OR economic model$.mp) AND Australia$.mp

Websites- accessed April 2005

| **Website** | **Address** |
| --- | --- |
| Medical Services Advisory Committee (MSAC) | [www.msac.gov.au/reports](http://www.msac.gov.au/reports) |
| Australian Government Department of Health and Ageing | [www.health.gov.au](http://www.health.gov.au/) |
| Health Insite- an Australian Government Initiative | [www.healthinsite.gov.au](http://www.healthinsite.gov.au/) |
| The Cancer Council Australia | [www.cancer.org.au](http://www.cancer.org.au/) |
| National Health and Medical Research Council | [www7.health.gov.au.nhmrc](http://www.health.gov.au.nhmrc/) |
| National Heart Foundation | [www.heartfoundation.com.au](http://www.heartfoundation.com.au/) |
| National Institute of Clinical Studies (NICS) | [www.nicsl.com.au](http://www.nicsl.com.au/) |
| ASERNIP-S | www.surgeons.org/asernip-s/ |
| Royal Australian college of General Practitioners | [www.racgp.org.au](http://www.racgp.org.au/) |
| Royal Australasian college of Physicians | [www.racp.edu.au](http://www.racp.edu.au/) |
| Royal Australasian College of Surgeons | [www.surgeons.org](http://www.surgeons.org/) |
| Australian Medical Association | [www.ama.com.au](http://www.ama.com.au/) |
| Joanna Briggs Institute | [www.joannabriggs.edu.au](http://www.joannabriggs.edu.au/) |
| Public Health Association of Australia | [www.phaa.net.au](http://www.phaa.net.au/) |
| Royal College of Nursing Australia | [www.rcna.org.au](http://www.rcna.org.au/) |
| ACT Health | www.health.act.gov.au/c/health |
| Health and Human Services Tasmania | [www.dhhs.tas.gov.au](http://www.dhhs.tas.gov.au/) |
| Queensland Health | [www.health.qld.gov.au](http://www.health.qld.gov.au/) |
| Northern Territory Department of Health and Community Services | [www.health.nt.gov.au](http://www.health.nt.gov.au/) |
| Department of Human Services Victoria | [www.dhs.vic.gov.au](http://www.dhs.vic.gov.au/) |
| New South Wales Health | [www.health.nsw.gov.au](http://www.health.nsw.gov.au/) |
| Department of Health- Government of Western Australia | [www.wa.health.gov.au](http://www.wa.health.gov.au/) |
| Department of Human Services South Australia | [www.dhs.sa.gov.au](http://www.dhs.sa.gov.au/) |
| Monash Institute for Health Services Research | www.med.monash.edu.au/healthservices |
| Department of Epidemiology and Preventative Medicine (DEPM) | [www.med.monash.edu.au/epidemiology](http://www.med.monash.edu.au/epidemiology) |
| Centre for Health Economices Research and Evalation (CHERE) | http://www.chere.uts.edu.au/ |
| Centre for Health Economics (CHE) | www.buseco.monash.edu.au/centres/che/ |
| School of Economics, Population Health, University of Queensland | www.uq.edu.au/economics/ |
| Program evaluation unit, University of Melbourne | www.peu.unimelb.edu.au/ |
| Medical Technology Assessment Group (M-TAG) | [www.m-tag.net](http://www.m-tag.net/) |
| ANU National Centre for Epidemiology and Population Health | http://nceph.anu.edu.au/ |
| Social and Public Health Economics Research Group (SPHERE) | http://www.sphere.curtin.edu.au/ |

Key author search

OVID Medline 1966 to present- searched April 2005

(Glasziou P$.au OR Hall J$.au OR Harris A$.au OR Carter R$.au OR Crowley S$.au OR Davey P$.au OR Cook J$.au OR Salkeld G$.au OR Segal L$.au OR Shiell A$.au OR Smith R$.au OR Butler J$.au) AND Australia$.mp
