# Supplementary material for: Review of Australian health economic evaluation – 245 interventions: what can we say about cost effectiveness?
Source: Cost Eff Resour Alloc. 2008 May 20;6:9. doi: 10.1186/1478-7547-6-9 (PMC2413209; doi:10.1186/1478-7547-6-9)
Supplement: Additional file 2 — APPENDIX 2_CERA. A copy of the second appendix which provides a list of the 245 Australian health interventions included in the systematic review along with an estimate of their cost effectiveness. [file 1478-7547-6-9-S2.doc]

APPENDIX 2 Cost effectiveness of 245 health interventions (reporting LY/QALY/DALY) in Australia

| **Intervention/patients/comparator** | **Cost per LY/QALY*** | **Year** | **Ref.** |
| --- | --- | --- | --- |
| **Heart disorders/ stroke** | | | |
| Enalapril maleate angiotensin-converting enzyme (ACE) inhibitor for congestive heart failure vs placebo | Dominant** | 1996 | 1. |
| Mediterranean diet- advised by cardiologist and dietician following myocardial infarction vs Western diet of AHA | Dominant | 2005 | 2. |
| Thrombolytic therapy given intravenously (for 1 hour) within 3 hours of stroke vs current practice | Dominant | 2004 | 3. |
| Mass media educational nutrition intervention with support materials and services for CHD prevention vs no intervention | $1,235 | 1999 | 4. |
| Hospital/rehabilitation nutrition intervention for those with CHD vs no intervention | $1,253 | 1999 | 4. |
| Ventillation-perfusion scintigraphy (VPS), leg ultrasound and pulmonary angioplasty for diagnosis of PE vs helical CT | $1,542 | 2000 | 5. |
| Community nutrition intervention for CHD high risk groups (low SES, aboriginal, other ethnic groups) ns no intervention | $1,639 | 1999 | 4. |
| GP nutrition intervention for high CHD risk individuals (cholesterol, BP, sedentary, obese) vs no intervention | $2,384 | 1999 | 4. |
| Aspirin therapy given within 48 hours of stroke daily for 2-4 weeks vs current practice | $2,924 | 2004 | 3. |
| Single bolus treatment with abciximab (Reo Pro) following PTCA multi-vessel disease vs placebo | $5,735 | 1998 | 6. |
| Single bolus treatment with abciximab (Reo Pro) following PTCA all patients vs placebo | $7,545 | 1998 | 6. |
| Single bolus treatment with abciximab (Reo Pro) following PTCA single vessel disease vs placebo | $11,947 | 1998 | 6. |
| Pravastatin statin lipid lowering pharmaceutical for those with ischaemic heart disease and average cholesterol vs placebo | $13,510 | 2002 | 7 |
| Pulmonary thromboendarterectomy for chronic thromboembolic pulmonary hypertension vs current medical management | $16,830 | 2001 | 8. |
| Ramipril ACE inhibitor pharmaceutical vs placebo | $21,405 | 2003 | 9. |
| Primary prevention with 40mg/day pravastatin for >5% 15 year risk of CHD mortality vs no pravastatin | $37,509 | 2001 | 10. |
| Helical CT scan, venous leg ultrasound and pulmonary angioplasty for diagnosis of suspected acute PE vs VPS | $39,050 | 2000 | 5. |
| Primary prevention with 40mg/day pravastatin for >2.5% 15 year risk of CHD mortality vs no pravastatin | $43,761 | 2001 | 10. |
| Primary prevention with 40mg/day pravastatin according to PBS criteria for risk of CHD vs no pravastatin | $123,155 | 2001 | 10. |
| Near patient cholesterol testing in general practice, private clinic or hospital clinic vs laboratory testing | $166,208 | 2001 | 11. |
| Video program plus written patient self-help materials in GP for adults with CVD risk factor/s vs routine care | $7,962,695 | 1997 | 12. |
| Video based lifestyle change program in GP for adults with CVD risk factor/s vs routine care | Dominated** | 1997 | 12. |
| **Diabetes / Impaired glucose tolerance** | | | |
| Group behavioural modification predominantly in the workplace for a group 10% IGT, 90%NGT vs usual care | Dominant | 1998 | 13. |
| Intensive lifestyle changes counselling and goal setting by GP and nurse for those with IGT vs standard lifestyle advice | Dominant | 2004 | 14. |
| Metformin- written information and script by GP for those with IGT vs standard lifestyle advice | Dominant | 2004 | 14. |
| Intensive lifestyle changes for those with IGT vs metformin | Dominant | 2004 | 14. |
| General practitioner advice for high risk adults with BMI>27 and CVD risks vs usual care | $1,432 | 1998 | 13. |
| Intensive diet and behavioural modification for women with previous GDM or IGT multidisciplinary team vs usual care | $1,718 | 1998 | 13. |
| Intensive diet and behavioural modification by a multidisciplinary team for a group 25%IGT 75%NGT vs usual care | $3,436 | 1998 | 13. |
| General practitioner advice for a group 10% IGT, 90%NGT vs usual care | $3,436 | 1998 | 13. |
| Intensive diet and behavioural modification by a multidisciplinary team for a group 10% IGT, 90%NGT vs usual care | $3,722 | 1998 | 13. |
| Reduced fat diet for IGT education, goal setting, group sessions and food diaries in community setting vs general diet advice | $11,311 | 2005 | 15. |
| GP NIDDM weight loss program- piggy backed on CVD intervention vs routine care | $15,033 | 1997 | 16. |
| Gastric bypass surgery plus prior counselling | $17,610 | 1998 | 13. |
| GP NIDDM weight loss program (using behavioural modification approach) independent vs routine care | $75,449 | 1997 | 16. |
| **Cancer** | | | |
| PCR diagnosis of patients gene rearrangement in acute myeloid leukaemia vs cytogenetic testing, FISH and prognosis | Dominant | 2003 | 17. |
| PCR in the diagnosis of patients with gene rearrangement in acute promyelocytic leukaemia vs cytogenetic testing | $409 | 2003 | 18. |
| Palliative radiotherapy for bone metastases vs no radiation | $1,632 | 2003 | 19. |
| PCR in the monitoring of patients with gene rearrangement in acute promyelocytic leukaemia vs cytogenetic testing | $7,981 | 2003 | 18. |
| Radiation therapy vs no radiation | $11,407 | 1995 | 20. |
| Exemestane- oral aromatase inhibitor for advanced breast cancer vs megestrol | $13,965 | 2002 | 21. |
| 5-fluorouracil and levamisole (chemotherapy) plus surgical resection for Dukes C colon cancer vs surgical resection alone | $32,239 | 1993 | 22. |
| rhTSH by IM injection prior to diagnostic scan for diagnosing recurrence or residual thyroid cancer vs standard THT | $66,097 | 2002 | 23. |
| Transurethral needle ablation (TUNA) benign prostatic hyperplasia vs transurethral resection of the prostate | Dominated | 2002 | 24. |
| **General Population** | | | |
| Media campaign with community support vs no program | Dominant | 1998 | 13. |
| Multi-media 2 fruit 5 veg campaign vs no campaign | $52 | 2005 | 15. |
| Australian mass-media tobacco campaign vs no campaign | $1,280 | 2005 | 15. |
| National skin cancer primary prevention campaign vs no campaign | $1,850 | 1999 | 25. |
| Massachusetts Tobacco Control Program- mass media campaign, local education etc vs no campaign | $2,328 | 2005 | 15. |
| FFFF- fighting fit fighting fat UK mass media campaign plus support material vs no campaign | $6,351 | 2005 | 15. |
| Nurse counselling in GP- 2 consults, tailored advice and goals vs education about fruit and vegetables | $11,881 | 2005 | 15. |
| Oxcheck- primary health care nurse checks UK vs no health checks | $14,197 | 2005 | 15. |
| Screening for melanoma by primary health physicians (every 5 years) for those over 50 years vs existing care | $20,850 | 1996 | 26. |
| Screening for melanoma by primary health physician every 2 years for those over 50 years vs screening every 5 years | $65,413 | 1996 | 26. |
| Exercise advice for the healthy elderly- individualised advice from physiologist re exercise plan vs nutrition pamphlet | $645,318 | 2005 | 15. |
| **Mental Health** | | | |
| Optimal treatment from clinical guidelines (inc.medication and psychosocial) for schizophrenia vs current treatment | Dominant | 2003 | 27. |
| Evidence based optimal care for anxiety disorders vs no treatment | Dominant | 2004 | 28. |
| Dexamphetamine pharmaceutical for ADHD vs current practice | $5,278 | 2004 | 29. |
| CBT (1 GP visit + 12 one hour consults) public psychologist for panic disorder vs current practice | $8,754 | 2004 | 30. |
| CBT (1 GP visit + 12 one hour consults) public psychologist for generalised anxiety disorder vs current practice | $8,883 | 2004 | 30. |
| Behavioural intervention in families (psychologist in clinic) for schizophrenia vs no family intervention | $10,299 | 2004 | 31. |
| CBT 14 weeks- public psychologist for major depression children and adolescents vs current practice | $11,586 | 2004 | 32. |
| Methylphenidate pharmaceutical for ADHD vs current practice | $19,310 | 2004 | 29. |
| Imipramine (tricyclinc antidepressant TCA)- oral daily dose for panic disorder vs current practice | $21,885 | 2004 | 30. |
| CBT (1 GP visit + 12 one hour consults) private psychologist for generalised anxiety disorder vs current practice | $25,747 | 2004 | 30. |
| Multiple family groups (delivered by senior psychologist in clinic) for schizophrenia vs no family intervention | $28,321 | 2004 | 31. |
| CBT (1 GP visit + 12 one hour consults) private psychiatrist for generalised anxiety disorder vs current practice | $29,609 | 2004 | 30. |
| CBT (1 GP visit + 12 one hour consults) public psychiatrist for generalised anxiety disorder vs current practice | $29,609 | 2004 | 30. |
| Venlafaxine (serotonin and noradrenalin reuptake inhibitors SNRI) for generalised anxiety disorder vs current practice | $29,609 | 2004 | 30. |
| SSRIs as 1st line treatment for major depression children and adolescents vs current practice | $29,609 | 2004 | 32. |
| SSRIs as 2nd line treatment for major depression children and adolescents vs no further treatment | $29,609 | 2004 | 32. |
| Clozapine oral pharmaceutical for treatment resistant schizophrenia with clear deterioration vs typical neuroleptics | $29,609 | 2005 | 33. |
| CBT (1 GP visit + 12 one hour consults) private psychologist for panic disorder vs current practice | $33,471 | 2004 | 30. |
| CBT (1 GP visit + 12 one hour consults) private psychiatrist for panic disorder vs current practice | $34,758 | 2004 | 30. |
| CBT 14 weeks private psychologist for major depression children and adolescents vs current practice | $36,045 | 2004 | 32. |
| CBT (1 GP visit + 12 one hour consults) public psychiatrist for panic disorder vs current practice | $38,620 | 2004 | 30. |
| Behavioural family management (psychologist in family home) for schizophrenia vs no family intervention | $38,620 | 2004 | 31. |
| CBT 14 weeks public psychiatrist for major depression children and adolescents vs current practice | $41,195 | 2004 | 32. |
| Cognitive behavioural therapy 14 weeks private psychiatrist for major depression children and adolescents | $43,769 | 2004 | 32. |
| Paroxetine (serotonin reuptake inhibitors SNRI) for panic disorder vs current practice | $48,919 | 2004 | 30. |
| Clozapine oral pharmaceutical for treatment resistant schizophrenia with little deterioration vs typical neuroleptics | $54,068 | 2005 | 33. |
| Risperidone oral pharmaceutical for schizophrenia vs typical neuroleptics | $61,792 | 2005 | 33. |
| Risperidone oral pharmaceutical for schizophrenia vs typical low-dose neuroleptics | $102,987 | 2005 | 33. |
| Olanzapine oral pharmaceutical for schizophrenia vs typical neuroleptics | $118,435 | 2005 | 33. |
| Optimal treatment from clinical guidelines (inc. medication and psychosocial for schizophrenia) vs no treatment | $137,499 | 2003 | 27. |
| Olanzapine oral pharmaceutical for schizophrenia vs risperidone | $205,974 | 2005 | 33. |
| Current treatment for schizophrenia vs no treatment | $250,827 | 2003 | 27. |
| **Vaccination** | | | |
| Haemophilus influenzae type b vaccination- 18 months single dose to non-Aboriginals vs no vaccination | $1,998 | 1994 | 34. |
| A/ Meningococcal vaccination for students years 10-12 and 1st yr uni high incidence regional popn vs no vaccination | $2,279 | 2001 | 35. |
| PRP-OMP vaccination at 2, 4 and 12 months for haemophilus influenzae type b disease vs vaccination only at 18 months | $3,189 | 1994 | 36. |
| Haemophilus influenzae type b vaccination- before 6 months three doses to non-Aboriginals vs no vaccination | $11,245 | 1994 | 34. |
| Haemophilus influenzae type b vaccination- 24-36 months catch up program to non-Aboriginals vs no vaccination | $14,004 | 1994 | 34. |
| Zanamivir prescribed in general practice to treat those at high risk of influenza vs current treatment | $14,470 | 2000 | 37. |
| Haemophilus influenzae type b vaccination- 12 months two doses to non-Aboriginals vs no vaccination | $14,825 | 1994 | 34. |
| Universal vaccination Hep B using combination vaccine at 2, 4, and 12 months vs selected vaccination of high risk infants | $15,270 | 2001 | 38. |
| Haemophilus influenzae type b vaccination- 37-48 months catch up program to non-Aboriginals vs no vaccination | $43,911 | 1994 | 34. |
| Both A and B vs no vaccination | $56,408 | 2001 | 35. |
| B/ Meningococcal vaccination of all year 12 students in a large popn (such as a state) vs no vaccination | $74,783 | 2001 | 35. |
| Both A and B vs A alone | $97,378 | 2001 | 35. |
| Pneumococcal conjugate vaccination- 4 doses at age 2,4,6 and 12-15 months of age vs no vaccination | $149,581 | 2004 | 39. |
| Strategy IV- varicella (chicken pox) vaccine catch up program adolescent and infant programs combined vs Strategy II | $151,411 | 2000 | 40. |
| Strategy II- varicella (chicken pox) vaccine at 12 months with MMR vs no vaccination | $161,389 | 2000 | 40. |
| Haemophilus influenzae type b vaccination- 49-60 months catch up program to non-Aboriginals vs no vaccination | $190,020 | 1994 | 34. |
| B vs A | $214,400 | 2001 | 35. |
| Universal TB screening all students Year 8 vs no screening | $216,158 | 2000 | 41. |
| Strategy III- -varicella (chicken pox) vaccine at 12 years with Hep B vs no vaccination | $218,528 | 2000 | 40. |
| Universal TB screening all students Year 1 vs no screening | $222,334 | 2000 | 41. |
| School based TB Mantoux screening targeted those born overseas year 1 vs universal screening year 1 | $321,149 | 2000 | 41. |
| School based TB Mantoux screening targeting those born overseas year 8 vs universal screening year 8 | $1,284,595 | 2000 | 41. |
| **Hepatitis/renal disorders** | | | |
| 65% Lamivudine (oral antiviral) and 12%IFN-alpha for chronic hepatitis B vs 20% IFN-alpha | Dominant | 2002 | 42. |
| 65% Lamivudine (oral antiviral) and 12%IFN-alpha for chronic hepatitis B vs no treatment | Dominant | 2002 | 42. |
| Valaciclovir prophylaxis (donor CMV positive /recipient negative) following renal transplant vs no prophylaxis | Dominant | 2004 | 43. |
| 20% IFN-alpha (parenteral administration 3 times/week) for chronic hepatitis B vs no treatment | $3,112 | 2002 | 42. |
| Interferon alfa for 6 months- injection self administered for hepatitis C vs no treatment, conventional management | $11,725 | 1999 | 44. |
| Interferon alfa for 12 months- injection self administered for hepatitis C vs IFN-alpha for 6 months | $18,369 | 1999 | 44. |
| Valaciclovir prophylaxis (recipient CMV positive) to prevent CMV infection following renal transplant vs no prophylaxis | $22,048 | 2004 | 43. |
| **Colorectal cancer** | | | |
| FOBT using Hemoccult test for population colorectal cancer screening biennial aged 55 to 74 years vs biennial age 55 | $3,814 | 2004 | 45. |
| Targeted general practice screening for colorectal cancer using FOBT biennial 55-74 years vs targeted GP screening 55-69 | $7,209 | 2004 | 46. |
| Targeted general practice screening for colorectal cancer using FOBT biennial 55-75+ years vs targeted GP screening 55-69 | $8,705 | 2004 | 46. |
| Targeted general practice screening for colorectal cancer using FOBT biennial 55-69 years vs status quo | $16,322 | 2004 | 46. |
| Annual faecal occult blood test (FOBT) for colorectal cancer ages 50-84 self administered vs current ad hoc screening | $18,360 | 2001 | 47. |
| 5 yearly colonoscopy for colorectal cancer for high risk, annual FOBT for rest vs current ad hoc screening | $18,724 | 2001 | 47. |
| 5 yearly colonoscopy for colorectal cancer by physician for medium risk, annual FOBT for rest vs current ad hoc screening | $20,430 | 2001 | 47. |
| Flexible sigmoidoscopy every 10 years for colorectal cancer prevention vs no screening | $20,891 | 2004 | 48. |
| Colonoscopy every 10 years for colorectal cancer prevention vs no screening | $23,980 | 2004 | 48. |
| FOBT using Hemoccult test for population colorectal cancer screening- biennial aged 55 to 74 years vs biennial age 55 | $25,894 | 2004 | 45. |
| Targeted general practice colorectal cancer screening using FOBT biennial 50-69 years vs targeted GP screening 55-69 | $32,644 | 2004 | 46. |
| Annual colorectal cancer screening faecal occult blood test (FOBT) vs no screening | $37,402 | 1996 | 49. |
| Biennial faecal occult blood test for colorectal cancer vs no screening | $51,209 | 2004 | 48. |
| Annual foetal occult blood test (FOBT) for colorectal cancer vs no screening | $58,318 | 2004 | 48. |
| Targetted general practice colorectal cancer screening using FOBT biennial 45-69 years vs targeted GP screening 55-69 | $68,009 | 2004 | 46. |
| **Drug and alcohol problems** | | | |
| Buprenorphine- oral narcotic given alternate days or every 3 days for heroin addiction vs methadone | Dominant | 2005 | 50. |
| Simple intervention (Saunders)- including family effects for problem drinking vs no intervention | $47 | 2005 | 51. |
| Brief intervention (Saunders)- including family effects for problem drinking vs no intervention | $68 | 2005 | 51. |
| Simple intervention- 5 minutes for problem drinking vs no intervention | $92 | 2005 | 52. |
| Brief intervention- 20 minutes for problem drinking vs no intervention | $133 | 2005 | 52. |
| Extended intervention (Saunders)- including family effects for problem drinking vs no intervention | $160 | 2005 | 51. |
| Brief intervention (Wilk)- including family effects for problem drinking vs no intervention | $283 | 2005 | 51. |
| Extended intervention- 120-150 mins over 4 sessions for problem drinking vs no intervention | $317 | 2005 | 52. |
| Brief intervention- motivational, self-help, 1-4 sessions, <1 hour each for problem drinking vs no intervention | $755 | 2005 | 52. |
| National drink-less alcohol brief intervention (5 mins GP advice) control-no support for GP vs no program | $877 | 2001 | 53. |
| Psychotherapy moderation-orientated cue exposure including family effects for alcohol vs behavioural self control training | $1,148 | 2005 | 51. |
| Psychotherapy motivational enhancement therapy including family effects for alcohol dependence vs no further counselling | $1,530 | 2005 | 51. |
| National drink-less alcohol brief intervention (5 mins GP advice) initial training vs no program | $1,664 | 2001 | 53. |
| Psychotherapy moderation-orientated cue exposure for alcohol dependence vs behavioural self control training | $2,414 | 2005 | 52. |
| National drink-less alcohol brief intervention (5 mins GP advice) maximal support for GP vs no program | $2,548 | 2001 | 53. |
| Psychotherapy motivational enhancement therapy (4 sessions/ 6 weeks) for alcohol dependence vs no further counselling | $3,789 | 2005 | 52. |
| Naltrexone- including family effects for detoxified severe physical dependence vs placebo | $5,843 | 2005 | 51. |
| Physician advice for smoking cessation minimal single consult 20 mins vs no intervention | $7,816 | 2005 | 54. |
| Physician advice for smoking cessation intensive >20 mins or materials additional to a leaflet vs no intervention | $9,748 | 2005 | 54. |
| Physician advice for smoking cessation- intensive in a high risk population vs minimal physician advice | $10,281 | 2005 | 54. |
| Bupropion plus counselling for smoking cessation vs placebo plus counselling | $13,778 | 2005 | 54. |
| Bupropion plus counselling for smoking cessation vs no intervention | $14,367 | 2005 | 54. |
| Naltrexone plus counselling for detoxified severe physical dependence vs placebo plus counselling | $14,595 | 2005 | 52. |
| Needle and syringe program for injecting drug users vs no program | $18,039 | 2002 | 55. |
| Physician advice for smoking cessation- intensive in an unselected population vs minimal physician advice | $18,204 | 2005 | 54. |
| Phone counselling on smokers helpline plus NRT vs no intervention | $22,437 | 2005 | 54. |
| Phone counselling on smokers helpline plus NRT vs NRT alone | $32,891 | 2005 | 54. |
| Phone counselling on smokers helpline plus NRT multiple sessions vs single sessions | $35,429 | 2005 | 54. |
| Psychotherapy- non-directive reflective listening 4 sessions over 6 weeks for alcohol dependence vs no further counselling | Dominated | 2005 | 52. |
| Psychotherapy- non-directive reflective listening- including family effects for alcohol dependence vs no further counselling | Dominated | 2005 | 51. |
| **Women’s health** | | | |
| Oestrogen therapy from 65 years (for remainder of life) vs no intervention | $15,659 | 1994 | 56. |
| Extending mammography screening to age 73 years vs no intervention | $16,196 | 2002 | 57. |
| Extending mammography screening to age 75 years vs no intervention | $18,084 | 2002 | 57. |
| Extending mammography screening to age 77 years vs no intervention | $20,165 | 2002 | 57. |
| Mammography screening- for those over 45 years vs no screening | $20,449 | 1992 | 58. |
| Oestrogen (hormone replacement therapy HRT) for women with symptoms after hysterectomy vs no intervention | $21,898 | 1992 | 59. |
| Extending mammography screening to age 79 years vs no intervention | $23,088 | 2002 | 57. |
| Oestrogen therapy from age 50 for life vs no intervention | $24,133 | 1994 | 56. |
| Oestrogen (hormone replacement therapy HRT) for women with symptoms and an intact uteri vs no intervention | $31,604 | 1992 | 59. |
| Mammographic screening 50-69, 2 yearly (I) vs II | $45,136 | 1993 | 60. |
| Tamoxifen- pharmaceutical for breast cancer prevention vs placebo | $47,272 | 2003 | 61. |
| Oestrogen and progesterone therapy for women with symptoms and an intact uteri vs no intervention | $53,738 | 1992 | 59. |
| Oestrogen therapy from 50 to 65 (15 years) vs no intervention | $54,898 | 1994 | 56. |
| Mammographic second yearly screening commencing age 40 vs secondly yearly screening commencing age 50 | $67,329 | 1997 | 62. |
| Mammogrpahic screening 40-49 2 yearly, 50-69 2 yearly (V) vs II | $73,450 | 1993 | 60. |
| Mammographic screening 40-49 2 yearly, 50-69 3 yearly (IV) vs II | $100,166 | 1993 | 60. |
| Mammographic screening 40-49 annually, 50-69 2 yearly (III) vs II | $110,806 | 1993 | 60. |
| Oestrogen (hormone replacement therapy HRT) for women with no symptoms after hysterectomy vs no intervention | $136,239 | 1992 | 59. |
| Dietary calcium and exercise for menopausal women vs no intervention | $174,825 | 1994 | 56. |
| Oestrogen and progesterone therapy for women with no symptoms and an intact uteri vs no intervention | $196,488 | 1992 | 59. |
| Oestrogen (hormone replacement therapy HRT) for women with no symptoms and an intact uteri vs no intervention | Dominated | 1992 | 59. |
| **Arthritis** | | | |
| Physiotherapy outpatients service (average of 8 consults) for rheumatoid arthritis vs no physiotherapy | $870 | 1992 | 63. |
| Physiotherapy outpatients service (average of 8 consults) for osteoarthritis vs no physiotherapy | $1,780 | 1992 | 63. |
| Glucosamine sulfate for osteoarthritis vs placebo | $3,862 | 2004 | 64. |
| Clinic based exercise in primary care for osteoarthritis vs usual care | $6,437 | 2004 | 64. |
| Topical capsaicin for osteoarthritis vs placebo | $6,437 | 2004 | 64. |
| Specially fitted knee brace for osteoarthritis vs usual care | $7,724 | 2004 | 64. |
| Total hip replacement for osteoarthritis vs usual care | $9,011 | 2004 | 64. |
| Clinic based exercise in outpatients for osteoarthritis vs usual care | $10,299 | 2004 | 64. |
| Primary care weight loss program for overweight or obese with osteoarthritis vs usual care | $14,161 | 2004 | 64. |
| Total knee replacement for osteoarthritis vs usual care | $14,161 | 2004 | 64. |
| Primary care weight loss program for overweight or obese with previous knee injury and osteoarthritis vs usual care | $15,448 | 2004 | 64. |
| Home based exercise intensive for osteoarthritis vs usual care | $19,310 | 2004 | 64. |
| Non-specific NSAIDs for osteoarthritis vs placebo | $19,310 | 2004 | 64. |
| Comprehensive mass media program for weight loss for osteoarthritis vs usual care | $25,747 | 2004 | 64. |
| Surgery for obese people for osteoarthritis vs usual care | $25,747 | 2004 | 64. |
| COX-2 specific NSAIDs for osteoarthritis vs placebo | $42,482 | 2004 | 64. |
| Lay-led group education for osteoarthritis vs usual care | Dominated | 2004 | 64. |
| Primary care educator plus phone support for osteoarthritis vs usual care | Dominated | 2004 | 64. |
| Home based exercise basic for osteoarthritis vs usual care | Dominated | 2004 | 64. |
| Knee arthroscopy with lavage for osteoarthritis vs usual care | Dominated | 2004 | 64. |
| COX-2 specific NSAIDs for osteoarthritis vs non-specific NSAIDs | Dominated | 2004 | 64. |
| Avocado/soy unsaponifiables for osteoarthritis vs placebo | Dominated | 2004 | 64. |
| **Musculoskeletal** | | | |
| Physiotherapy outpatients service (average of 8 consults) post back surgery vs no physiotherapy | $1,253 | 1992 | 63. |
| Physiotherapy outpatients service (average of 8 consults) for tendon injuries/dislocations vs no physiotherapy | $1,358 | 1992 | 63. |
| Physiotherapy outpatients service (average of 8 consults) for neck pain vs no physiotherapy | $1,671 | 1992 | 63. |
| Physiotherapy outpatients service (average of 8 consults) for overuse injuries vs no physiotherapy | $2,045 | 1992 | 63. |
| Physiotherapy outpatients service (average of 8 consults) for lower back pain vs no physiotherapy | $2,120 | 1992 | 63. |
| Physiotherapy outpatients service (average of 8 consults) post fracture vs no physiotherapy | $6,048 | 1992 | 63. |
| Physiotherapy outpatients service (average of 8 consults) for sprains and strains vs no physiotherapy | $6,470 | 1992 | 63. |
| Low intensity ultrasound (LIUS) self administered at home for fresh tibia fracture vs placebo | $133,284 | 2001 | 65 |
| Low intensity ultrasound (LIUS) self administered at home for fresh radius fracture vs placebo | $627,277 | 2001 | 65. |
| Low intensity ultrasound (LIUS) self administered at home for scaphoid fracture vs placebo | $801,521 | 2001 | 65. |
| **Obesity** | | | |
| Intensive diet and behavioural modification- multidisciplinary team for seriously obese and IGT vs usual care | Dominant | 1998 | 66. |
| Group behavioural modification- predominantly workplace for overweight and obese men vs usual care | Dominant | 1998 | 66. |
| Lifestyle change to prevent type 2 diabetes- physician and nutritionist for those overweight and IGT vs general diet advice | $2,115 | 2005 | 15. |
| The Victorian Active Script Programme- GPs prescribe exercise for sedentary adults vs no program | $4,961 | 2004 | 67. |
| Gastric bypass surgery plus prior counselling for seriously obese vs usual care | $6,586 | 1998 | 66. |
| Exercise sessions for sedentary elderly- range of exercise activities available in community vs no organised sessions | $17,616 | 2005 | 15. |
| Gutbusters- 6 week workplace course- education and weight loss for overweight males vs no program | $22,283 | 2005 | 15. |
| Physical activity counselling in GP for inactive adults vs usual care | $32,668 | 2005 | 15. |
| Massecheutts 10 school intervention- 16 lessons on TV, activity and diet vs no program | $56,383 | 2005 | 15. |
| Orlistat pharmaceutical plus diet for obesity vs placebo plus diet | $94,197 | 2005 | 15. |
| **Antenatal / post natal care** | | | |
| Exogenous surfactant- given with assisted ventilation for extremely low birthweight infants (500-999g) vs no surfactant | Dominant | 1997 | 68. |
| Positive pressure assisted ventilation for extremely immature infants (24-28 weeks gestation) vs no assisted ventilation | $225 | 1989 | 69. |
| Intensive/special care nursery within an existing hospital for high risk/ low birthweight infants vs no intensive nursery | $2,185 | 1989 | 70. |
| Rh D immunoglobulin- post partum only (mother Rh- with no Anti D in blood) vs no Anti-D | $4,998 | 1999 | 71. |
| Universal antenatal screening for HIV vs | $23,457 | 2004 | 72. |
| Rh D immunoglobulin (Anti-D)-antenatal with indications (AWI) + post partum (PP) vs PP only | $28,134 | 1999 | 71. |
| Rh D immunoglobulin (Anti-D)-antenatal prophylaxis (AP) + post partum vs AP and AWI and PP | $40,471 | 1999 | 71. |
| Rh D immunoglobulin (Anti-D)-antenatal prophylaxis (at 28 and 34 weeks) + post partum vs AWI and PP | $44,865 | 1999 | 71. |
| Rh D immunoglobulin (Anti-D)-antenatal prophylaxis + antenatal with indications + post partum vs AP and PP | $60,315 | 1999 | 71. |
| **Miscellaneous** | | | |
| Laparoscopic cholecystectomy minimal access surgery to remove gallbladder vs open cholecystectomy | Dominant | 1994 | 73. |
| Extracorporeal shock wave lithotripsy (ESWL) non-invasive day procedure for gallstones vs open cholecystectomy | Dominant | 1994 | 73 |
| Misoprostol prophylaxis for NSAID induced gastrointestinal damage in patients with peptic ulcer history vs no misoprostol | $7,988 | 2000 | 74. |
| Cochlear implant 3 day hospital stay for surgery to implant for children vs no implant | $11,345 | 1999 | 75. |
| Cochlear implant 3 day hospital stay for surgery to implant for profoundly deafened adults vs no implant | $27,960 | 1999 | 75. |
| Cochlear implant 3 day hospital stay for surgery to implant for partially deafened adults vs no implant | $44,250 | 1999 | 75. |
| Misoprostol prophylaxis for NSAID induced gastrointestinal damage in patients over 65 years vs no misoprostol | $51,583 | 2000 | 74. |
| Misoprostol prophylaxis for NSAID induced gastrointestinal damage all patients vs no misoprostol | $53,558 | 2000 | 74. |
| Recombinant factor VIIa (NovoSeven) home treatment IV push injection for haemophiliac children vs previous usual care | $70,094 | 2001 | 76. |
| Low osmolar contrast media for radiocontrast studies for patients at high risk of adverse reactions only vs high osmolar | $111,705 | 1991 | 77. |
| Low osmolar contrast media for radiocontrast studies for low risk patients vs high osmolar | $348,553 | 1991 | 77. |
| Laparoscopic cholecystectomy minimal access surgery to remove gallbladder vs ESWL | $3,615,412 | 1994 | 72. |

*Standardised to 2005 A$

** For the purposes of the data analysis dominant interventions were assigned a value of $0 and dominated interventions were assigned a value of $1,000,000

†This piece of work was in press at the time of the review (April 2005) but has since been published and the reference is provided

References

| 1 | Butler et al. Aust NZ J Med 1996;26:89-95 | 27 | Andrews et al. Br J Psychiatry 2003;183:427-35 | 53 | Wutzke et al. Soc Sci Med 2001;52:863-70 |
| --- | --- | --- | --- | --- | --- |
| 2 | In press† (Dalziel et al. Journal of Nutrition 2006;136:1879-85) | 28 | Issakidis et al. Psych Med 2004;34:19-35 | 54 | Segal et al. Centre for Health Economics, 2005 Research Paper 5 |
| 3 | Moodie et al. Stroke 2004;35:1041-6 | 29 | Donnelly et al. Aust NZ J Psychiatry 2004;38:592-601 | 55 | Department of Health and Ageing 2002 |
| 4 | Van Gool et al. CHERE 1999 Project Report 11 | 30 | Heuzenroeder et al. Aust NZ J Psychiatry 2004;38:602-12 | 56 | Geelhoed et al. Aust J Public Health 1994;18:153-60 |
| 5 | Larcos et al. Aust NZ J Med 2000;30:195-201 | 31 | Mihalopoulus et al. Aust NZ J Psychiatry 2004;38:511-19 | 57 | Barratt et al. MJA 2002;176:266-272 |
| 6 | Arstides et al. Heart 1998;79:12-17 | 32 | Haby et al. Aust NZ J Psychiatry 2004; 38:579-91 | 58 | Hall et al. Soc Sci Med 1992;34:993-1004 |
| 7 | Glasziou et al. MJA 2002;21:420-6 | 33 | Magnus et al. Aust NZ J Psychiatry 2005;39:44-54 | 59 | Cheung et al. MJA 1992;156:312-16 |
| 8 | MSAC 2001 application 05 | 34 | McIntyre et al. Aust J Public Health 1994;18:394-400 | 60 | Carter et al. Aust J Public Health 1993;17:42-50 |
| 9 | Smith et al. Intern Med J 2003;33:414-19 | 35 | Skull et al. Int J Epidemiol 2001;30:571-8 | 61 | Eckermann et al. Aust NZ J Public Health 2003;27:34-40 |
| 10 | Lim et al. MJA 2001;175:459-64 | 36 | Harris et al. MJA 1994;160:483-8 | 62 | Irwig et al. NHMRC National Breast Cancer Centre, 1997 |
| 11 | MSAC 2001 application 1026 | 37 | Mauskopf et al. Pharmacoeconomics 2000;17:611-20 | 63 | Haas et al. CHERE 1992, Discussion paper 6 |
| 12 | Salkeld et al. Health Policy 1997;41:105-19 | 38 | Harris et al. Aust NZ J Public Health 2001;25:222-9 | 64 | Segal et al. MJA 2004;180:S11-17 |
| 13 | Segal et al. Health Promotion Int 13:197-209 | 39 | Butler et al. Vaccine 2004;22:1138-49 | 65 | MSAC 2001 application 1030 |
| 14 | Palmer et al. Clinical Therapeutics 26:304-21 | 40 | Scuffham et al. Vaccine 2000;18:407-15 | 66 | Segal et al. Health Promotion Int 1998;13:197-209 |
| 15 | Segal et al. Centre for Health Economics, 2005 Research Paper 1 | 41 | Lowin et al. Aust NZ J Public Health 2000;24:247-53 | 67 | Sims et al. Bri J Sports Med 2004;38:19-25 |
| 16 | Dalton et al. CHPE 1997 Working paper 65 | 42 | Crowley et al. J Gastroent Hepatol 2002;17:153-64 | 68 | The Victorian Infant Collaborative Study Group. J Paediatr Child Health 1997;33:202-8 |
| 17 | MSAC 2003 application 9a(iii) | 43 | Tolden et al. Clin Transplant 2004;18:312-20 | 69 | Doyle e tal. MJA 1989;150:558-68 |
| 18 | MSAC 2003 application 9a(ii) | 44 | Shiell et al. MJA 1999;171:189-93 | 70 | Tudehope et al. Aust Paediatr J 1989;25:61-5 |
| 19 | Barton et al. Radiation Oncology 2003;47:274-78 | 45 | MSAC 2004 application 18 | 71 | NHMRC, 1999 |
| 20 | Barton et al. Clinical Oncology 1995;7:287-92 | 46 | Stone et al. Aust and NZ J Public Health 2004;28:273-82 | 72 | Graves et al. Journal of Inf Dis 2004;190:166-74 |
| 21 | Lindgren eta l. Pharmacoeconomics 2002;20:101-8 | 47 | Stevenson. Australian Digital Thesis Program 2001 | 73 | Cook et al. Health Econ 1994;3:157-68 |
| 22 | Smith et al. MJA 1993;158:319-22 | 48 | OLeary et al. J Gastroenterol and Hepatol 2004;19:38-47 | 74 | Davey et al. Pharmacoeconomics 2000;17:295-04 |
| 23 | MSAC 2002application 1043 | 49 | Salkeld et al. Aust NZ J Public Health 1996;20:138-43 | 75 | Carter et al. Int J HTA 1999;15:520-30 |
| 24 | MSAC application 1014 | 50 | Harris et al. Pharmacoeconomics 2005 23:77-91 | 76 | Ekert et al. Haemophilia 2001;7:279-85 |
| 25 | Carter et al. Health Promotion International 1999;14:73-82 | 51 | In press† (Mortimer et al. Alcohol & Alcoholism 2006;41(1):92-98) | 77 | Henry et al. MJA 1991;154:766-72 |
| 26 | Girigis et al. J Med Screen 1996;3:47-53 | 52 | In press† (Mortimer et al. Alcohol & Alcoholism 2006;40(6):549-555) |  |  |
